# Fibrinogen plasma concentration is an independent marker of haemodynamic impairment in chronic thromboembolic pulmonary hypertension

**DOI:** 10.1038/srep04808

**Published:** 2014-04-28

**Authors:** Jan K. Hennigs, Hans Jörg Baumann, Nicole Lüneburg, Gesine Quast, Lars Harbaum, Jan Heyckendorf, Karsten Sydow, Bernhard Schulte-Hubbert, Michael Halank, Hans Klose

**Affiliations:** 1 Centre for Pulmonary Hypertension; 2 II. Department of Internal Medicine; 3 Institute of Clinical Pharmacology and Toxicology and Cardiovascular Research Centre; 4 Department of General and Interventional Cardiology, Hamburg University Heart Centre, University Medical Centre Hamburg - Eppendorf, Hamburg, Germany; 5 I. Department of Internal Medicine, Carl Gustav Carus University Hospital, Dresden, Germany; 6 These authors contributed equally to this work.; 7 Current address: Vera Moulton Wall Pulmonary Vascular Research Laboratories and Cardiovascular Institute, Stanford University - School of Medicine, Stanford, California, USA

## Abstract

Fibrinogen has a crucial role in both inflammation and coagulation, two processes pivotal for the pathogenesis of pulmonary hypertension. We therefore aimed to investigate whether fibrinogen plasma concentrations a) are elevated in pulmonary arterial hypertension (PAH) and chronic thromboembolic pulmonary hypertension (CTEPH) and b) may serve as a novel biomarker for haemodynamic impairment. In a dual-centre, retrospective analysis including 112 patients with PAH (n = 52), CTEPH (n = 49) and a control cohort of patients with suspected PAH ruled out by right heart catheterisation (n = 11), we found fibrinogen plasma concentrations to be increased in patients with PAH (4.1 ± 1.4 g/l) and CTEPH (4.3 ± 1.2 g/l) compared to control patients (3.4 ± 0.5 g/l, p = 0.0035 and p = 0.0004, respectively). In CTEPH patients but not in PAH patients fibrinogen was associated with haemodynamics (p < 0.036) and functional parameters (p < 0.041). Furthermore, fibrinogen was linked to disease severity (WHO functional class, p = 0.017) and independently predicted haemodynamic impairment specifically in CTEPH (p < 0.016). Therefore, fibrinogen seems to represent an important factor in CTEPH pathophysiology and may have the potential to guide clinical diagnosis and therapy.

Chronic thromboembolic pulmonary hypertension (CTEPH) is one of the most frequent causes of pulmonary hypertension (PH). Following the current understanding, CTEPH results as a complication in 0.1–9.1% of the patients with acute pulmonary embolism (APE) from an obstruction of predominantly proximal pulmonary arteries with organized blood clots^1^.

Evaluation of data from large registries identified a set of risk factors for CTEPH including ventriculo-atrial shunts, infected pacemaker leads, splenectomy, previous venous thromboembolism, thyroid hormone replacement therapy and a history of malignancy^1^,^2^. Although dysregulation of thrombosis and thrombolysis is observed in a subgroup of CTEPH patients^3^ this mechanism does not fully explain the pathophysiology. Important classical thromboembolic risk factors are lacking and in about 25% of the CTEPH patients APE occurred asymptomatically^1^. Hence, the mechanistic view of CTEPH as a consequence of central pulmonary vessel obstruction appears to be too simplistic^1^.

Pulmonary arterial hypertension (PAH), another main PH group, shares several features indistinguishable from CTEPH: histopathology of peripheral vascular lesions^4^, acute vasoreactivity testing^1^, extent of endothelial dysfunction (ED)^5^ and fibrin resistance to lysis^1^,^3^,^6^. However, it is still unknown which exact pathophysiological mechanisms separate these two diseases from each other and in which areas the diseases can be seen as one entity.

Increase of pulmonary vascular resistance in PAH is related to different mechanisms, including vasoconstriction, loss of distal arteries, proliferative and obstructive remodelling of the pulmonary vessel wall, microthrombosis, and inflammation. The latter mechanism gained attention as elevated concentrations of inflammatory markers such as interleukin (IL)-1, IL-6, IL-13, and tumour necrosis factor-alpha were detected in PAH patients plasmas and tissues^7^. Recently, C-reactive protein (CRP), representing the most established clinical marker of inflammation, was shown to be a prognostic factor in PAH patients^8^. To which extent inflammation is relevant for CTEPH pathogenesis is uncertain.

The plasmatic glycoprotein fibrinogen is both marker of inflammation and central player in the coagulation cascade^9^ characterised by its interaction with thrombin leading to conversion of fibrinogen to fibrin by limited proteolysis. Fibrin-stabilising-factor then causes cross-linkage of fibrin-molecules and consequently thrombus formation. Fibrinogen is induced by IL-6 as part of the acute phase reaction^10^. Interestingly, fibrinogen concentrations have recently been shown to be elevated in a small cohort of PAH patients^11^ and elevated fibrinogen plasma concentrations are considered as risk factor for coronary heart disease^12^. However, the diagnostic or predictive potential of fibrinogen has not yet been assessed in PH.

In the present study we aimed to (a) investigate whether fibrinogen plasma concentrations are elevated in patients with PAH and/or CTEPH, (b) characterise possible differences between patients with PAH and CTEPH regarding fibrinogen plasma concentrations and (c) evaluate a potential association of fibrinogen plasma concentrations with established risk factors for disease progression and severity in both groups.

## Results

### Baseline characteristics of study population

A total of 112 patients were included in this study - 52 (46%) patients with PAH, 49 (44%) with CTEPH and 11 (10%) control individuals with suspected pulmonary hypertension that was eventually ruled out by right heart catheterisation (RHC). PAH patients comprised idiopathic PAH (n = 39), familial PAH (n = 3) and PAH associated with portal hypertension (n = 6), connective tissue disease (n = 2) and congenital heart disease (n = 2).

Baseline demographics, hemodynamic, inflammatory and disease severity parameters of the study population are given in Table 1. Clinical baseline parameters did not differ between PAH and CTEPH subgroups except gender and height. PAH and CTEPH patients had severe disease as reflected by highly elevated mean pulmonary artery pressure (mPAP), mean pulmonary vascular resistance (PVR) and a World Health Organisation function class (WHO FC) of III or IV in the majority of the patients (88% and 85%, respectively). PAH and CTEPH groups did not differ regarding hemodynamic status or functional class. Median follow-up was similar in both groups: 33 months (range 4–111) in the PAH and 34 months (range 1–108) in the CTEPH subgroup.

### Fibrinogen plasma concentrations are elevated in PAH and CTEPH at time of diagnosis

At time of initial RHC patients with PAH or CTEPH showed significantly elevated fibrinogen plasma concentrations (PAH: 4.1 ± 1.4 g/l, p = 0.0035; CTEPH: 4.3 ± 1.2 g/l, p = 0.0004) compared to non-PH control patients (3.4 ± 0.5 g/l, mean ± standard deviation).

**Figure 1:**
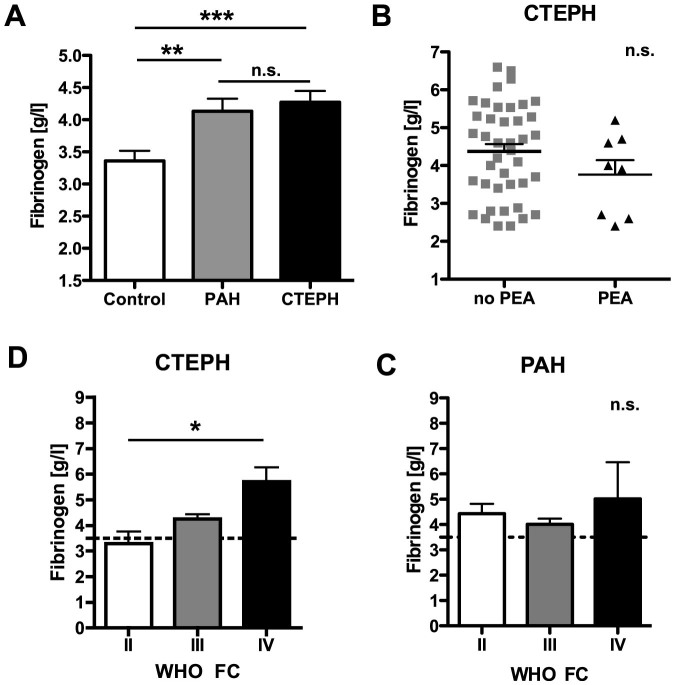
Fibrinogen plasma concentrations at time of right heart catheterisation. (A) At time of right heart catheterisation (RHC) fibrinogen plasma concentrations varied significantly in patients with pulmonary arterial hypertension (PAH) or chronic thromboembolic pulmonary hypertension (CTEPH) when compared with non-PH control individuals (Control: mean [fibrinogen] 3.4 ± 0.5 g/l, n = 11; PAH: mean [fibrinogen] 4.1 ± 1.4 g/l, n = 52, p = 0.0035; CTEPH: mean [fibrinogen] 4.3 ± 1.2 g/l, n = 49, p = 0.0004, ANOVA). No statistically significant differences occurred between patients with PAH and CTEPH (p = 0.59, ANOVA). (B) Patients that underwent pulmonary endarterectomy in the latter course did not show significantly different fibrinogen concentrations at time of RHC (p = 0.20, two-sided Student's t-test). WHO functional class increased with fibrinogen concentration only in CTEPH (C, p = 0.0172, ANOVA) but not in PAH patients (D, p = 0.52, ANOVA). Plasma concentrations are expressed as mean ± S.D. *** indicates p-values <0.001, ** indicates p < 0.01, * indicates p < 0.05, n.s.: non significant (p > 0.05).

No significant differences in fibrinogen concentrations were detected between PAH and CTEPH (Table 1 and Fig. 1A). Eight CTEPH patients (16%) underwent pulmonary endarterectomy (PEA) following the initial RHC. Fibrinogen concentrations from CTEPH patients undergoing PEA in the later course did not differ from inoperable CTEPH patients (Fig. 1B).

### Fibrinogen plasma concentrations correlate with clinical and haemodynamic parameters in CTEPH but not in PAH

In the control group, fibrinogen plasma concentrations only correlated with age and N-terminal fragment of pro-brain natriuretic peptide (NT-proBNP) concentrations (Table 2, left columns). In PAH patients, fibrinogen plasma concentrations only correlated with CRP concentrations but not with demographic, haemodynamic or disease severity parameters (Table 2, middle columns).

Intriguingly, in CTEPH patients fibrinogen plasma concentrations were correlated with haemodynamic measures (right atrial pressure, RAP; mPAP, PVR, and cardiac index, CI) and disease severity markers (NT-proBNP, 6-minute walk distance, 6-MWD, arterial partial pressure of CO_2_, PaCO_2_) but not with demographic data (age or gender; Table 2, right columns).

In addition, Analysis of Variances (ANOVA) revealed a gradually increasing association between WHO functional class and fibrinogen concentrations in CTEPH (WHO FC II 3.3 ± 0.4, WHO FC III 4.3 ± 0.2; WHO FC IV 5.7 ± 0.5 g/l, p = 0.0172) but not in PAH patients (Fig. 1C+D).

### Fibrinogen plasma concentrations predict haemodynamic impairment in CTEPH patients in univariate and multivariate regression analyses

Linearity of fibrinogen plasma concentration with clinical and haemodynamic parameters was tested by regression analysis. In univariate analysis fibrinogen concentrations were significantly associated with RAP, mPAP, PVR, CI, and 6-MWD (Table 3, left columns).

To test independence of fibrinogen plasma concentrations from demographical data and disease severity a multivariate analysis including age, sex, WHO functional class, fibrinogen and the hemodynamic and functional parameters identified in univariate analysis (RAP, mPAP, PVR, CI, and 6-MWD) was performed. Here, fibrinogen was confirmed as an independent marker of haemodynamic impairment as RAP and mPAP were independently associated with fibrinogen concentrations (Table 3, right columns). In addition, PVR and CI showed a non-significant trend to be associated with fibrinogen concentrations.

### Fibrinogen is a predictor of CTEPH in patients at risk

**Figure 2:**
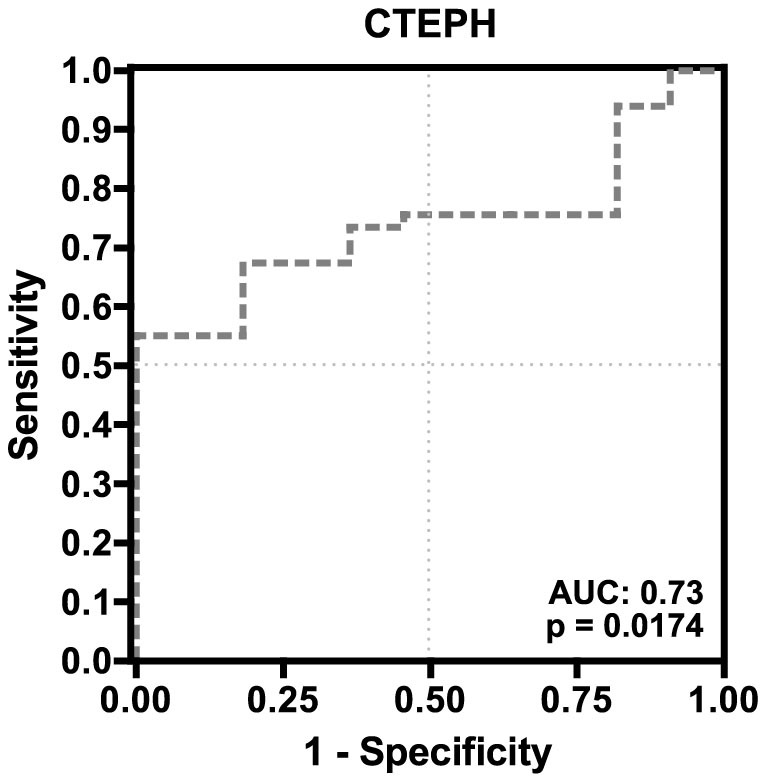
Receiver-Operator-Characteristics for fibrinogen in CTEPH patients.

A receiver operator characteristics (ROC) curve analysis was performed to identify a cut-off fibrinogen plasma concentration that might serve as diagnostic biomarker in CTEPH (Fig. 2).

On that account a cut-off value of 3.65 g/l would provide 82% specificity (95% CI: 48.2–97.7%) and 65% sensitivity (95% CI: 49.5–77.8%) for the test. Odds ratio using this cut-off would be 8.2 (95% CI: 1.59–42.4) for detection of CTEPH at time of RHC.

Clinical significance of this cut-off value was further tested by contingency analysis of haemodynamics, clinical characteristics and fibrinogen plasma concentrations. Fibrinogen plasma concentrations above 3.65 g/l were strongly associated with unfavourable haemodynamic parameters including mPAP (p = 0.001), PVR (p = 0.004), RAP (p = 0.007) and central venous oxygen saturation (SvO_2_, p = 0.006). Furthermore, a fibrinogen concentration >3.65 g/l was also linked with elevated CRP concentrations (p = 0.023, Table 4).

## Discussion

In the present study we found that fibrinogen plasma concentrations were elevated both in patients with PAH and CTEPH compared to non-PH patients at risk. We could show that in CTEPH patients, but not in PAH patients, fibrinogen plasma concentrations were correlated with markers of disease severity, haemodynamics and CRP concentrations (as a surrogate marker for inflammation). Most importantly, fibrinogen plasma concentrations represent an independent predictor of impaired haemodynamics in CTEPH. Moreover, ROC analysis for detection of at-risk CTEPH patients showed a high specificity with a moderate sensitivity.

Despite numerous studies investigating coagulation parameters, we revealed elevated fibrinogen plasma concentrations in CTEPH patients for the first time. Huber et al. found fibrinogen concentrations to be increased in PAH and “secondary” PH as opposed to healthy controls and patients with PH related to Eisenmenger's reaction^13^. In contrast, Welsh et al. detected increased fibrinogen concentrations only in patients with associated forms of precapillary PH but not in idiopathic PAH^14^. Interestingly, fibrinogen concentrations correlated with mPAP in the combined population of their study. In our study, we found elevated fibrinogen concentrations in both PAH and CTEPH patients compared to non-PH controls. However, the independent association with haemodynamic parameters (RAP, mPAP) was only found in CTEPH patients. In contrast to all existing studies, our control group was not recruited from healthy individuals but from patients with suspected PH. Despite diseases with known substantial inflammatory activity within the control cohort – i.e. coronary artery disease and systemic sclerosis –, we were able to reveal significantly different fibrinogen concentrations between the control group and PAH or CTEPH patients (regardless of fibrinogen being considered as an acute phase protein).

Our observations raise the question why fibrinogen concentrations are elevated in PAH and CTEPH: First, fibrinogen may be a marker indicating a pro-coagulant condition. Second, fibrinogen may be elevated in response to inflammatory activity in PAH/CTEPH, which could be initiated by a systemic or localized inflammation within the pulmonary vascular bed. Third, fibrinogen *per se* may be a central mediator of inflammation leading to vascular remodeling in CTEPH.

The first hypothesis is supported by findings that elevated fibrinogen concentrations were associated with increased thrombus formation and blood viscosity in PH patients^15^. Also, when obtained from CTEPH patients, fibrinogen is known to be relatively resistant to *in vitro* proteolysis by plasmin^16^ and a specific fibrinogen Aα polymorphism increased the risk of developing CTEPH by strengthening the fibrinogen chain cross-linkage^17^. Fibrinogen's pathological impact on coagulation in CTEPH was further substantiated by a high prevalence of 5 different dysfibrinogenaemias (fibrinogen_San Diego I-V_) among patients with CTEPH. All 5 dysfibrinogenaemias seem to confer resistance to fibrinolysis thereby fostering CTEPH development^18^.

A review of established CTEPH risk factors corroborated a potential role of fibrinogen in coagulation-dependent CTEPH pathogenesis. These risk factors included pacemakers^19^, splenectomy^20^, non-0 blood type^21^, lupus anticoagulant^22^, thyroid replacement therapy/subacute hypothyroidism^23^, malignancy^24^ and age^10^.

The hypothesis of a pivotal role of inflammation in PH pathogenesis is underlined by various studies investigating circulating inflammatory markers. i.e. IL-1 and IL-6, perivascular infiltrates and tertiary lymphoid tissues in PAH patients^7^. Intriguingly, inflammation seems to play a role in CTEPH pathogenesis, too, since markers of inflammatory activity (tumour necrosis factor (TNF)-alpha and monocyte chemoattractant protein-1, MCP-1) were found to be elevated in CTEPH and decreased after PEA^25^,^26^. Interestingly, MCP-1 concentrations were higher in arterial compared with mixed venous blood suggesting increased production or reduced clearance within the pulmonary vasculature^27^.

Differences in biomarkers of inflammation and coagulation may help to distinguish PAH from CTEPH. Apart from our findings regarding fibrinogen concentrations, differences between PAH and CTEPH were found for IL-6, CRP, soluble CD-40-ligand and fractalkine^8^,^25^,^27^. All these data confirm that both diseases, despite the significant overlap, may have distinct pathophysiology. CRP, for instance, was found to be elevated both in PAH and CTEPH^8^,^25^,^27^, but was only correlated with outcome in PAH and not in CTEPH patients.

However, Wynants and colleagues also report increased proliferative, coagulatory and chemotactic activity of primary pulmonary artery endothelial (PA-EC) and smooth muscle cells (PA-SMC) from CTEPH patients after stimulation with CRP^28^. In our population we were able to confirm a correlation between CRP and fibrinogen concentrations suggesting a similar cross talk between inflammation and coagulation.

In addition, chronic exposure of PA-EC and PA-SMC to fibrinogen induces altered cytosolic calcium responses, which are likely to confer to vascular remodelling^29^. Furthermore, fibrinogen directly enhanced vasoconstriction through increased endothelin-1 release from PA-EC^30^ and indirectly via its degradation products fibrinopeptide A and B^31^.

Indeed, it has been shown that fibrinogen depletion not only diminished inflammatory responses but also myocardial remodelling in an ischemia-reperfusion model^32^. These observations support the hypothesis that fibrinogen might not only be a marker of inflammation in the studied processes but seems to contribute to vascular remodelling *per se*.

In the present study, however, we only had access to CRP concentrations as a single, rather unspecific marker of inflammation and cannot proof a causative link between fibrinogen and inflammation or vascular remodelling in CTEPH.

Nevertheless, our study reflects “real-life” data from two independent German PH referral centres (with a relatively small sample size). We could detect independent associations between fibrinogen plasma concentrations and impaired haemodynamic parameters in CTEPH patients. It is important to note that in our study PAH and CTEPH patients also did not differ regarding the extent of haemodynamic impairment. Therefore, a bias due to different disease severities was minimized, which was often not the case in previous studies.

A possible reason why we did not detect an association between fibrinogen concentrations and parameters of disease severity in PAH patients might be due to the heterogeneity of the underlying pathologies associated with PAH, e.g. scleroderma or HIV. Due to the small number of patients within each subgroup we were unable to identify a statistical relation of this aspect. In addition, fibrinogen plasma concentrations are influenced by a plethora of lifestyle and demographic factors such as gender, age, body weight, physical activity, smoking or stress^10^. In order to minimize these influences, we matched our study population with regard to age, sex and body weight. Moreover, anticoagulatory therapy was not administered in a controlled fashion. However, since vitamin K-antagonists do not seem to influence fibrinogen plasma concentrations, we do not believe that this issue provided a relevant bias to our data^10^,^33^. Actually, heparin (that was used routinely before RHC in our patients) even seems to decrease fibrinogen plasma concentrations^34^. On the contrary, due to the small sample size and lack of an independent evaluation/follow-up cohort, we cannot rule out an overestimation of fibrinogen's role as a predictor of impaired haemodynamics in CTEPH. Additional limitations of the study include its retrospective nature and a low mortality within our CTEPH population, which made a statistical correlation of fibrinogen to survival impossible. Also, other coagulatory parameters (e.g. D-dimers or prothrombin fragments) that might have helped to distinguish between a thromboembolic and inflammatory nature of increased fibrinogen plasma concentrations were not available for our analysis.

In summary, fibrinogen plasma concentrations are elevated in PAH and CTEPH patients compared to control patients at time of initial RHC. In CTEPH but not in PAH patients increased fibrinogen concentrations predict impaired haemodynamic parameters and clinical severity. This correlation, which is supported by experimental data from other groups in PA-EC and PA-SMC signalling, together with the fact that established risk factors for CTEPH seem to be associated with elevated fibrinogen concentrations, indicate a central role of fibrinogen in the pathophysiology of CTEPH. Therefore, we strongly believe that fibrinogen is a hitherto unrecognized diagnostic and disease severity marker in CTEPH, which warrants further experimental and clinical studies.

## Methods

### Study population

At the two participating German centres (University Medical Centre Hamburg-Eppendorf, Hamburg, and Carl Gustav Carus University Hospital, Dresden) the attending physicians retrospectively evaluated all patients presenting to the pulmonary hypertension clinics between January 2001 and January 2010 (Dresden) or November 2011 (Hamburg) for inclusion into this study.

Inclusion criteria were as follows: 1. >18 years of age, 2. Proven pre-capillary pulmonary hypertension as determined by RHC with an mPAP of ≥25 mm Hg in combination with pulmonary artery wedge pressure (PAWP) of ≤15 mm Hg, 3. Clinical classification into WHO groups I or IV following current guidelines^35^, and 4. Determination of fibrinogen plasma concentrations at time of catheterisation (within 3 days around the procedure). In all patients, lung ventilation and perfusion scintigraphy was used in addition to thoracic computed tomography.

Patients belonging to Dana Point/WHO group II, III or V, patients with documented haematological disorders or significant coagulopathies including liver failure or elevated liver enzymes > 2.5-times upper normal limit were excluded. Anticoagulation was switched from coumadin to low-molecular-weight heparin prior to right heart catheterisation. PEA was offered to all patients with CTEPH who were deemed eligible candidates. During the same time frame a control group was generated from patients referred to the Hamburg centre with suspected PH that was eventually ruled out during the course of clinical workup (n = 11 patients). These cases were selected intentionally as control patients and should not be considered as healthy individuals as they reported symptoms severe enough to justify right heart catheterization in the first place.

All data analysed within this study were obtained during clinical routine, de-identified and retrospectively transferred into an anonymised database before evaluation. No additional testing or procedures were performed for this study. Local law (Hamburgisches Krankenhausgesetz, HmbKHG, §12, 1–3 and §12a, 1–5) regulates the use of patient data in accordance with the Declaration of Helsinki and specifically approves the use of anonymised data for retrospective analyses without individual informed consent thereby superseding Institutional Review Board approval. This study was conducted in full accordance with the principles and requirements of the Declaration of Helsinki and HmbKHG.

### Clinical parameters

Charts of patients were retrieved and data concerning demographics, Dana Point/WHO classification, WHO functional class (FC), 6-MWD, right ventricular systemic pressure (RVSP), CI, mPAP, RAP, PVR, systemic vascular resistance (SVR), SvO_2_, *P*_a_CO_2_, fibrinogen plasma concentrations, CRP concentrations, NT-proBNP concentrations, observation time and mortality was extracted wherever possible.

### Processing of blood samples

At time of visit for RHC, all blood samples except for SvO_2_ (obtained during RHC) and *P*_a_CO_2_ (arterial or capillary blood gas analysis) were obtained by venipuncture of a peripheral vein during routine clinical and diagnostic workup. All samples were analysed immediately. For fibrinogen assessment, blood was drawn into standardised 3.1% (v/v) trisodium citrate tubes at a final citrate to blood ratio of 1:10 according to manufacturer's instruction (Sarstedt, Nümbrecht, Germany) and processed using a fully automated haemostasis analyser (BCS XP, Siemens Healthcare, Erlangen, Germany) with the Multifibren U reagent kit (Siemens Healthcare, Erlangen, Germany). Reference values for fibrinogen plasma concentrations within this study were 1.8–3.5 g/l.

### Statistical analyses

Statistical analyses were performed using SPSS 17.0 (SPSS Inc.; Chicago, Illinois USA) and GraphPad Prism 5.0 (GraphPad Software Inc.; La Jolla, California USA) from an anonymous database. Normally distributed continuous variables (Kolmogorov–Smirnov test) were expressed as mean ± SD unless otherwise indicated. Differences between continuous variables were analysed using unpaired t-test with Welch's correction. Differences between two nominal variables were analysed using the chi-square test. ANOVA was used to analyse differences between more than two groups. Associations between variables were assessed by Pearson and Spearman correlation coefficients, respectively. Variables, which significantly correlated with plasma fibrinogen concentrations were further analysed in an univariate linear regression model at first. Multivariate linear regression analysis with forced entry selection of the covariates age, sex and WHO FC was constructed afterwards. Two-sided values of p < 0.05 were considered statistically significant.

## Figures and Tables

**Table 1:** Baseline characteristics of included patients at time of right heart catheterisation (n = 112)

	PAH§	CTEPH	Control	P value
*n = , % or mean ± SD*	*n = , % or mean ± SD*	*n = , % or mean ± SD*		
No. of patients	52	49	11	-
Follow-up [months]	39.0 ± 27.6	39.7 ± 32.2	-	-
Age [years]	62.5 ± 10.0	62.4 ± 11.9	59.8 ± 11.6	a 0.47^b^ 0.51^c^ 0.99
Sex Male	13	23	4	a 0.50^b ^0.54^c^ *0.02*
Female	39	26	7	
Body weight [kg]	72.1 ± 11.0	78.0 ± 16.1	73.5 ± 15.1	a 0.87^b^ 0.61^c^ 0.052
Height [cm]	164 ± 9.1	170 ± 9.3	168 ± 9.8	a 0.48^b^ 0.72^c^ *0.003*
WHO FC I	0 (0%)	1 (2%)	1 (25%)	0.043*
II	6 (12%)	6 (13%)	1 (25%)	
III	42 (84%)	39 (81%)	2 (50%)	
IV	2 (4%)	2 (4%)	0 (0%)	
RVSP [mm Hg]	71.8 ± 10.2	79.1 ± 8.1	41.0#	^a/b ^n.d.^c^ 0.59
mPAP [mm Hg]	44.8 ± 11.3	45.2 ±13.9	13.2 ± 3.1	^a/b^*<0.0001*^c ^0.86
PAWP [mm Hg]	8.8 ± 7.2	7.1 ± 0.7	7.3 ± 2.7	a 0.28^b^ 0.87^c ^0.21
RAP [mm Hg]	4.0 ± 2.6	5.7 ± 0.9	-	^a/b^ n.d.^c ^0.14
PVR [dyne·s/cm^5^]	809.6 ± 363.6	836.1 ± 61.5	105.7 ± 27.4	^a/b^*<0.0001*^c^ 0.90
CI [l/min·m^2^]	2.2 ± 0.6	2.1 ± 0.1	2.6 ± 0.3	a 0.11^b ^0.08^c ^0.41
SvO_2_ [%]	65.5 ± 2.0	63.4 ± 1.4	-	^a/b^ n.d.^c^ 0.40
6-MWD[m]	351.7 ± 94.2	321.7 ± 19.8	550.0#	^a/b ^n.d.^c^ 0.21
*P*_a_CO_2_ [kPa]	4.4 ± 0.5	4.5 ± 1.0	5.5 ± 1.1	a *0.001*^b^ *0.034*^c^ 0.32
NT-proBNP [ng/l]	3106.0 ± 4661.1	2388.0 ± 2784.6	240.2 ± 173.8	a *0.0011*^b^*<0.0001*^c^ 0.43
C-reactive protein [mg/l]	11.9 ± 30.3	9.3 ± 9.9	5.9 ± 3.2	a 0.22^b ^0.07^c^ 0.60
Fibrinogen [g/l]	4.1 ± 1.4	4.3 ± 1.2	3.4 ± 0.5	a *0.0035*^b ^*0.0006*^c^ 0.65

a = PAH vs. Control, ^b^ = CTEPH vs. Control, ^c^ = PAH vs. CTEPH, n.d. : not determinable; * χ^2^ analysis;

#single value; statistically significant p values are given in *Italics*. ^§^See text for subclasses and abbreviations.

**Table 2:** Correlation of plasma fibrinogen concentrations with risk factors, haemodynamic parameters and markers of inflammation in patients with PAH, CTEPH and control individuals

	Control		PAH		CTEPH	
Parameter	R	P value	R	P value	R	P value
Age	0.817	**0.002**	0.158	0.273	0.068	0.650^a^
Sex	0.233	0.491	0.128	0.367	0.051	0.732^a^
WHO FC	0.638	0.362	−0.006	0.967	0.172	0.247^a^
RVSP			−0.081	0.897	0.373	0.189^b^
mPAP	0.487	0.128	0.044	0.760	0.373	**0.009**^a^
RAP	-	-	−0.065	0.734	0.621	**<0.0001**^b^
PVR	0.200	0.580	0.105	0.513	0.326	**0.035**^a^
CI	−0.766	0.445	−0.178	0.272	−0.322	**0.033**^a^
SvO_2_	-	-	0.022	0.913	−0.236	0.160^a^
6-MWD	-	-	−0.235	0.125	−0.347	**0.038**^a^
*P*_a_CO_2_	−0.150	0.810	−0.201	0.279	−0.331	**0.040**^a^
NT-proBNP	0.821	**0.045**	−0.136	0.442	0.492	**0.002**^b^
C-reactive protein	0.480	0.160	0.712	**<0.001**	0.492	**0.0009**^b^

^a^Pearson correlation coefficient.

^b^Spearman correlation coefficient.

See text for abbreviations.

**Table 3:** Univariate and multivariate regression analyses of fibrinogen plasma concentrations and haemodynamic and disease severity markers in CTEPH patients

	Univariate analysis†			Multivariate analysis*		
Variable	R^2^	Beta	P value	R^2^	Beta	P value
RAP	0.244	2.309	**0.002**	0.269	2.186	**0.008**
mPAP	0.137	4.284	**0.011**	0.254	4.047	**0.015**
PVR	0.100	105.581	**0.041**	0.194	99.049	*0.055*
CI	0.096	−0.127	**0.043**	0.362	−0.098	*0.076*
6-MWD	0.113	−31.569	**0.048**	0.435	−21.129	0.124
*P*_a_CO_2_	0.096	−0.129	0.058	0.113	−0.115	0.137

^†^Univariate analysis includes plasma fibrinogen concentration and specific variable.

*Multivariate analysis includes plasma fibrinogen concentration, age, gender and WHO functional class plus specific variable. For abbreviations see text.

**Table 4:** Association of fibrinogen plasma concentrations with PAH risk factors, haemodynamic and inflammatory parameters in CTEPH patients*

			Fibrinogen			
Parameter	Variable	n =	<1.8 g/l [%]	1.8 - 3.65 g/l [%]	>3.65 g/l [%]	P value
mPAP [mm Hg]	>45.2^#^	23	0.0	13.0	87.0	0.001
	<45.2	25	0.0	56.0	44.0	
PVR [dyne*s*cm^−5^]	>836.1^#^	19	0.0	15.8	84.2	0.004
	<836.1	24	0.0	58.3	41.7	
RAP [mm Hg]	>12	6	0.0	0.0	100.0	0.007
	<12	32	0.0	50.0	50.0	
CI [l/min x m^2^]	>2.0	21	0.0	52.4	47.6	0.072
	<2.0	23	0.0	26.1	73.9	
SvO_2_ [%]	>70	7	0.0	85.7	14.3	0.006
	<70	30	0.0	30.0	70.0	
*P*_a_CO_2_ [mm Hg]	<35	24	0.0	29.2	70.8	0.057
	>35	15	0.0	60.0	40.0	
6-MWD [m]	<250	11	0.0	18.2	81.8	0.080
	>250	25	0.0	48.0	52.0	
C-reactive protein [mg/l]	>5.0	20	0.0	55.0	45.0	0.023
	<5.0	23	0.0	21.7	78.3	
WHO FC	I/II	7	0.0	32.5	67.5	0.220
	III/IV	40	0.0	57.1	42.9	
NT-proBNP [ng/l]	>150	35	0.0	25.0	75.0	0.623
	<150	4	0.0	37.1	62.9	

*Deviations from total are due to missing data; **^#^**Variables were arbitrarily dichotomized by their arithmetical means as marker to distinguish disease severity.

See main text for abbreviations.
